# Immunohistochemical analysis of ADAMTS-1, versican and pEGFR expressions in periapical granuloma and radicular cyst

**DOI:** 10.1186/s12903-021-01462-x

**Published:** 2021-03-06

**Authors:** Nádia Marielly Gomes Batista, Antonia Taiane Lopes de Moraes, Karolyny Martins Balbinot, Osvaldo Rodrigues de Souza Neto, Juliana Melo da Silva Brandão, Maria Sueli da Silva kataoka, Sérgio de Melo Alves Júnior, João de Jesus Viana Pinheiro

**Affiliations:** 1 Federal University of Pará Brazil, Avenue Augusto Corrêa, 01, Belém, PA 66075-110 Brazil; 2 Department of Oral Pathology, School of Dentistry, Federal University of Pará, Avenue Augusto Corrêa, 01, Belém, PA 66075-110 Brazil; 3 Cell Culture Laboratory, Faculty of Dentistry, Federal University of Pará, Rua Augusto Corrêa, 01, Guamá, Belém, PA 66075110 Brazil

**Keywords:** Radicular cyst, Periapical granuloma, ADAMTS1 protein, Versicans, Immunohistochemistry

## Abstract

**Background:**

ADAMTS expression can be associated with several inflammatory processes, and has been correlated with tumorigenesis of some neoplasms, but its participation in the development of periapical lesions has not been investigated. Therefore, our objective was to verify the expression of ADAMTS-1, versican and pEGFR in Periapical Granuloma (PG) and in the Radicular Cyst (RC) since they are the most common lesions of the periapex.

**Methods:**

25 samples of RC and 10 of PG were used. As a control, 10 samples of inflammatory fibrous hyperplasia (IFH) and 10 of dental follicle (DF) were used. The expression of these proteins was investigated using immunohistochemistry.

**Results:**

In the epithelium of RC, IFH and DF, the expression of ADAMTS-1 was greater in DF than in RC (p < .001). Versicano showed greater expression in IFH than in RC, DF than in RC (p < .001). pEGFR showed greater expression in IFH and RC than in DF (p < .01 and p < .05, respectively). In connective tissue, ADAMTS-1 expression was greater in PG and RC than in IFH and DF (p < .001). Versicano showed greater expression in PG, RC and IFH compared to DF (p < .001). In pEGFR there was a higher expression in PG when compared to RC, IFH and DF (p < .001). Greater immunostaining occurred in the RC than in the DF (p < .001).

**Conclusions:**

Our results suggest that the studied proteins may participate in the pathogenesis of PG and RC, through the interaction of these proteins, in the remodeling of the ECM (versican) by ADAMTS-1, producing bioactive fragments, which could activate EGFR, contributing to the formation, growth and maintenance of injuries.

## Background

Periapical granuloma (PG) consists of a chronically inflamed granulation tissue, which is the most common periapical lesion, representing about 70 % of all lesions in the periapex [1]. Radicular cysts (RC) are odontogenic lesions of inflammatory origin and constitute 52–68 % of all cysts in the jaw [2]. It is believed that RC formation involves the proliferation of Malassez’s epithelial rests and the formation of epithelial chains that grow and fuse into a three-dimensional spherical mass. The tissues trapped within this epithelial mass gradually degenerate due to the loss of blood and oxygen supply (hypoxia condition) and lead to the formation of the cystic cavity [3].

The etiopathogenic mechanisms of these lesions are still not well understood; however, the interaction between inflamed tissue and cellular components indicates the relevance of molecular pathways in their origin/development [4]. The matrix metalloproteinases (MMPs), which break down collagen and bone matrix, may also contribute to RC growth [5]. The proteolysis of the extracellular matrix (ECM) has been widely related to ADAMTS (A Disintegrin and Metalloproteinase with Thrombo Spondin Motifs) endopeptidases [6].

The ADAMTS-1, which was identified as the first protease of the ADAMTS family, is physiologically expressed in the organism and participates in several biological processes [7]. It is suggested that dental pulp cells (odontoblasts, cementoblasts, cementocytes, osteoblasts, osteocytes and periodontal ligament cells) regulate the expression of ADAMTS-1, ADAMTS-4, ADAMTS-5 and versican substrate [8].

ADAMTS-1 can degrade proteoglycans, among which the versican figures as one of the most biologically important [9, 10]. Versican is a member of the chondroitin sulfate proteoglycan family and is involved in both normal and pathological cell proliferation [11, 12]. The proteolytic cleavage of versican forms bioactive fragments such as G3 domain with epidermal growth factor (EGF)-like motifs that can bind to EGFR (epidermal growth factor receptor) and promote cell proliferation [13, 14]. In summary, the degradation of versican by ADAMTS-1 in an inflammatory environment could release bioactive fragments, activate the EGFR and lead the proliferation of Malassez’s epithelial rests.

ADAMTS-1 expression may be associated with several inflammatory processes and has been correlated with tumorigenesis of some neoplasms [5], however, its role in the development of periapical lesions has not yet been investigated. Considering that the interaction mechanism among ADAMTS-1, versican and phosphorylated epidermal growth factor receptor (pEGFR) can influence the etiopathogenesis of periapical lesions, this study aimed to investigate the immunohistochemical expression of ADAMTS-1, versican and pEGFR in RC and PG.

## Methods

### Sample selection

Under the approval of the Ethics Committee for Human Research of the Federal University of Pará (CEP-ICS/UFPA-3.630.466), 55 paraffin-embedded tissue blocks from different individuals were gathered from the Laboratory of Pathological Anatomy and Immunohistochemistry (School of Dentistry, UFPA, Belém, Brazil). These tissue samples were previously microscopically diagnosed as RC (n = 25), PG (n = 10). Samples of inflammatory fibrous hyperplasia (IFH) (n = 10) and dental follicle (DF) (n = 10) were used as control.

### Immunohistochemistry

Sections with 3-mm thickness were obtained from the paraffin-embedded tissue blocks, mounted on silanized slides, deparaffinized, washed with xylol and dehydrating ethanol solution. Subsequently, the samples were immersed in 3% hydrogen peroxide and methanol (1:1) to block endogenous peroxidase activity. Antigenic recovery was performed with citrate buffer (pH 6.0) in a Pascal pressure chamber (Dako Cytomation, Carpinteria, CA, USA) for 30 s at 125 ºC. After treatment with 1 % bovine serum albumin (Sigma-Aldrich, St. Louis, MO, USA) in phosphate-buffered saline for 1 h, the sections were incubated for 1 h in a humid chamber at room temperature with the primary antibodies anti-ADAMTS-1 (Abcam, Cambridge, MA, USA), anti-versican (Sigma-Aldrich) e anti-pEGFR (Sigma-Aldrich) diluted at 1:50 and incubated separately. Then, the slides were incubated and treated at room temperature with a dextran polymer–based complex (Reveal; Spring Bioscience, Pleasanton, CA), and diaminobenzidine (DAB) was used as a chromogenic agent (Liquid DAB + Substrate, Spring Bioscience®). Finally, the slides were counterstained with Mayer’s hematoxylin (Sigma-Aldrich) and assembled with mounting medium (Permount, Fisher Scientific, Fair Lawn, NJ, USA). Samples of intraductal breast carcinoma were used as a positive control. The negative control was obtained by omitting the primary antibody, which was replaced by non-immune serum.

### Evaluation

In each sample, 5 images were randomly acquired by a microscope (AxioScope, Carl Zeiss, Oberkochen, Germany) equipped with a color camera (AxioCam HRC, Carl Zeis) at 40x magnification. The areas immunostained with DAB were selected with a specific plugin of imaging software (Colour Deconvolution, ImageJ, NIMH, Bethesda, MD, USA) and the fraction areas (%) were measured. The data was used to determine:

- the differences of ADAMTS-1, versican and pEGFR expressions in the epithelial tissue between RC and controls;
- the differences of ADAMTS-1, versican and pEGFR expressions in the connective tissue among RC, PG and controls;
- the correlation between the expression of ADAMTS-1, versican and pEGFR in each lesion.

### Statistical analysis

Since the data presented a non-normal distribution evidenced by the Shapiro-Wilk test, differences between groups were assessed by the Kruskal–Wallis test followed by Bonferroni’s multi-comparison post hoc test a significance level of 95% (p < .05) (Graph Pad Prism 5.0, San Diego, CA, USA).

## Results

**Fig. 1:**
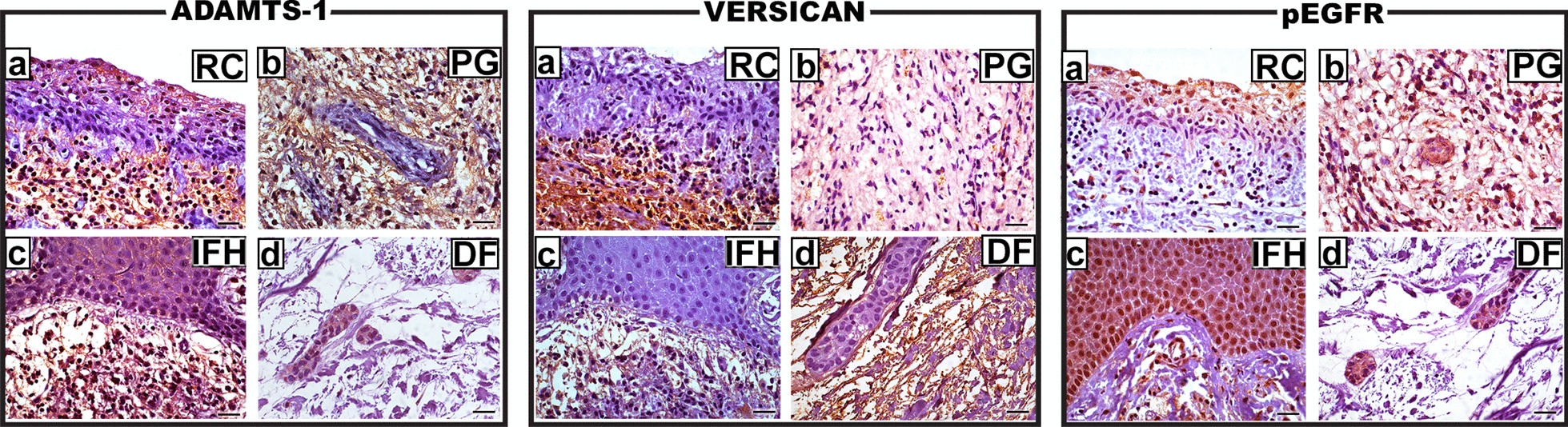
Representative photomicrographs of the immunohistochemical expressions of ADAMTS-1, versican and pEGFR in RC (Reveal, **a** ×630), PG (Reveal, **b** ×630), IFH (Reveal, **c** ×630) and DF (Reveal, **d** ×630). The expressions of ADAMTS-1 and versican were predominantly granular and cytoplasmic, while pEGFR was greatly expressed in the cytoplasm and cell membrane

ADAMTS-1, versican and pEGFR were expressed in every sample of all lesions (Fig. 1). The expressions of ADAMTS-1 and versican were predominantly granular and cytoplasmic, while pEGFR was greatly expressed in the cytoplasm and cell membrane.

**Fig. 2:**
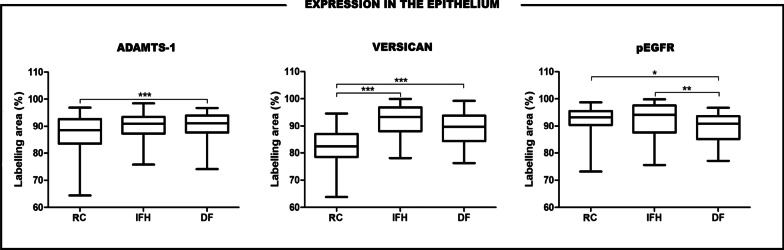
Expressions of ADAMTS-1, versican and pEGFR in the epithelium of RC, PG, IFH and DF. Horizontal lines represent the median, bars indicate the 25–75 % percentile distribution, and vertical lines indicate the 10–90 % percentile (***Kruskal–Wallis test: p < .001; **Kruskal–Wallis test: p < .01; *Kruskal–Wallis test: p < .05)

Considering the epithelial tissue, DF showed a significantly higher expression of ADAMTS-1 than RC (p < .001). RC presented a significantly lower expression of versican in comparison to IFH and DF (p < .001). In contrast, IFH and RC showed a significantly higher pEGFR expression than DF (p < .01 and p < .05, respectively) (Fig. 2; Table 1).

**Fig. 3:**
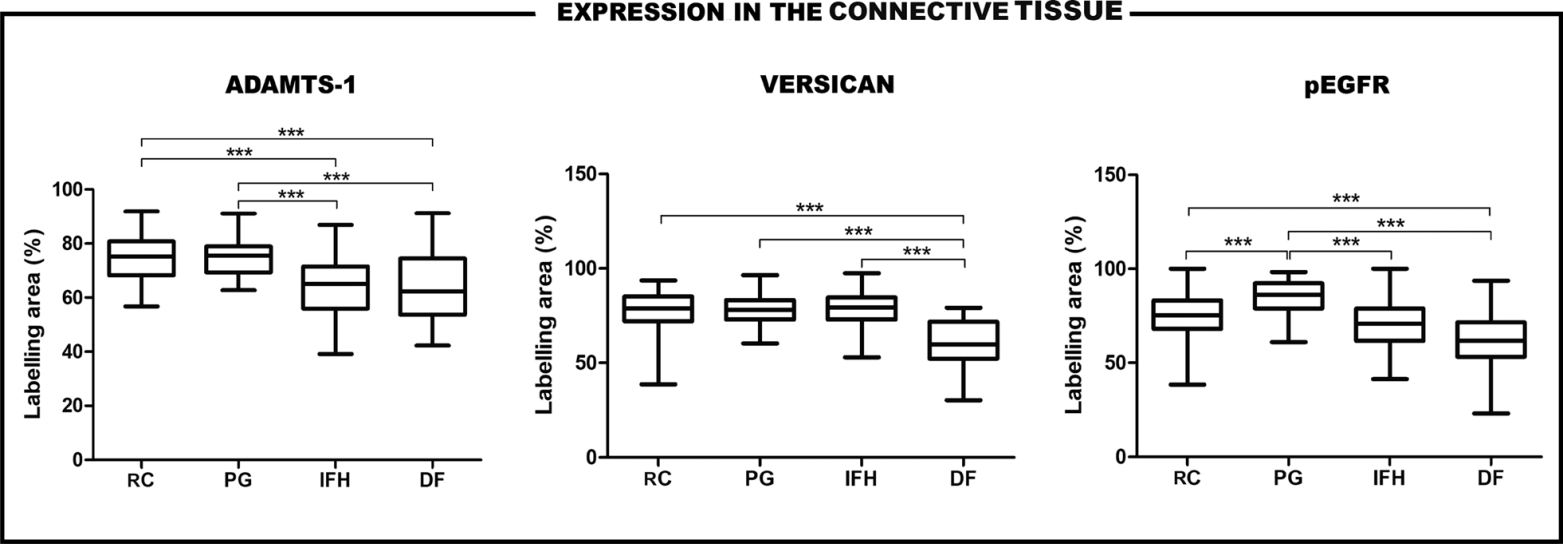
Expressions of ADAMTS-1, versican and pEGFR in the connective tissue of RC, PG, IFH and DF. Horizontal lines represent the median, bars indicate the 25–75 % percentile distribution, and vertical lines indicate the 10–90 % percentile (***Kruskal–Wallis test: p < .001)

In the connective tissue, the ADAMTS-1 expression was significantly higher in PG and RC when compared to IFH and DF (p < .001). Inflammatory lesions (PG, RC and IFH) presented a significantly higher expression of versican in comparison to DF (p < .001). The pEGFR expression in PG was significantly higher than in RC, IFH and DF (p < .001); in addition, pEGFR expression was significantly higher in RC when compared to DF (p < .001) (Fig. 3; Table 2).

## Discussion

Among the proteoglycans that can be degraded by ADAMTS-1, versican plays an important role in inflammatory lesions since it binds to a wide variety of receptors and other inflammatory components and regulates their availability and activity [13]. ADAMTS-1 can also interact with growth factors and activate EGFR, which contributes to cell proliferation, migration and angiogenesis [5–7].

The mechanisms that stimulate the proliferation of epithelial cell rests are not completely elucidated. (Lin et al. 1996) reported that RC exhibited higher EGFR expression than PG [15]. Thus considering that EGFR is present in the Malassez’s epithelial rests at the apical periodontal ligament [16], the activation of these receptors could induce several biochemical events, such as signal transduction, activation of protein kinases, phosphorylation of regulatory proteins, gene transcription and protein synthesis. Such events would lead to epithelial cell proliferation and potential cyst formation in periapical lesions of endodontic origin [17].

The present study demonstrated that both RC and PG expressed ADAMTS-1, versican and pEGFR. Moreover, ADAMTS-1 expression has been found in alveolar bone and periodontal ligament, ECM remodeling, morphogenesis/embryogenesis, follicular development and ovulation, cyclic endometrial remodeling, angiogenesis, as well as in the development of cancer, thrombotic and inflammatory conditions [8, 18].

In this study, ADAMTS-1 expression in the epithelial tissue of DF was higher than RC; however, in the connective tissue, inflammatory lesions (PG, RC and IFH) presented higher ADAMTS-1 expression than DF, which corroborates with other studies [19, 20]. It was previously reported that inflammatory cells and growth factors (inflammatory cytokines, IL-1 and TNF-α) around the inflamed connective tissue capsule stimulate proliferation and angiogenesis of the epithelial lining of the lesion and that the presence of MMPs in RC would figure as an additional pathway for cystic growth due to both collagen and bone matrix breakdown [19]. Therefore, it seems that the interactions between inflamed tissue and cell component represent an important lesion growth pathway and that ADAMTS-1 may have an influence on this mechanism due to ECM degradation.

Our findings in connective tissue indicated higher versican expression in PG, RC and IFH when compared to DF. In the epithelium, versican was highly expressed in both DF and IFH than RC. This expression profile associated with a proliferative cell phenotype such as embryonic tissues was previously reported by Wight [13]. One study revealed mRNA versican expression in the outer enamel epithelium of mice tooth germs [21]. Therefore, versican expression may be associated to some extent with the development of odontogenic cysts, which originate from embryonic tissues of the dental organ.

The pEGFR expression in the inflammatory cells of the lesions investigated in this study corroborates with other studies such as Song et al. 2016, which found that EGFR activation is directly related to the chronic inflammatory process in asthma [22]. Isidro et al. 2015 reported that the activation of the SP/NK-1R/EGFR signaling pathway is associated with increased colonic inflammation [23]. Our findings in the epithelium revealed lower pEGFR expression in the epithelium of DF when compared to RC and to the other control lesion (IFH). Li et al. 1997 reported that the EGFR expression level in the epithelial lining of the three most common types of odontogenic cysts (odontogenic keratocysts, dentigerous cysts and RC), follicular and unicystic ameloblastomas and PG seems to relate to the presence of inflammation in the adjacent connective tissue instead of epithelial cell proliferation [24].

It was previously demonstrated that EGFR is involved in cell proliferation induced by G3 domain of versican and the effects on cell proliferation were mediated by EGF-like motifs [12]. The authors showed that the deletion of EGF-like motifs significantly reduced versican’s ability to stimulate NIH3T3 cells proliferation, which suggests that EGF-like motifs play a role in the enhancement of cell growth due to an interaction with the EGFR on the cell surface.

**Fig. 4:**
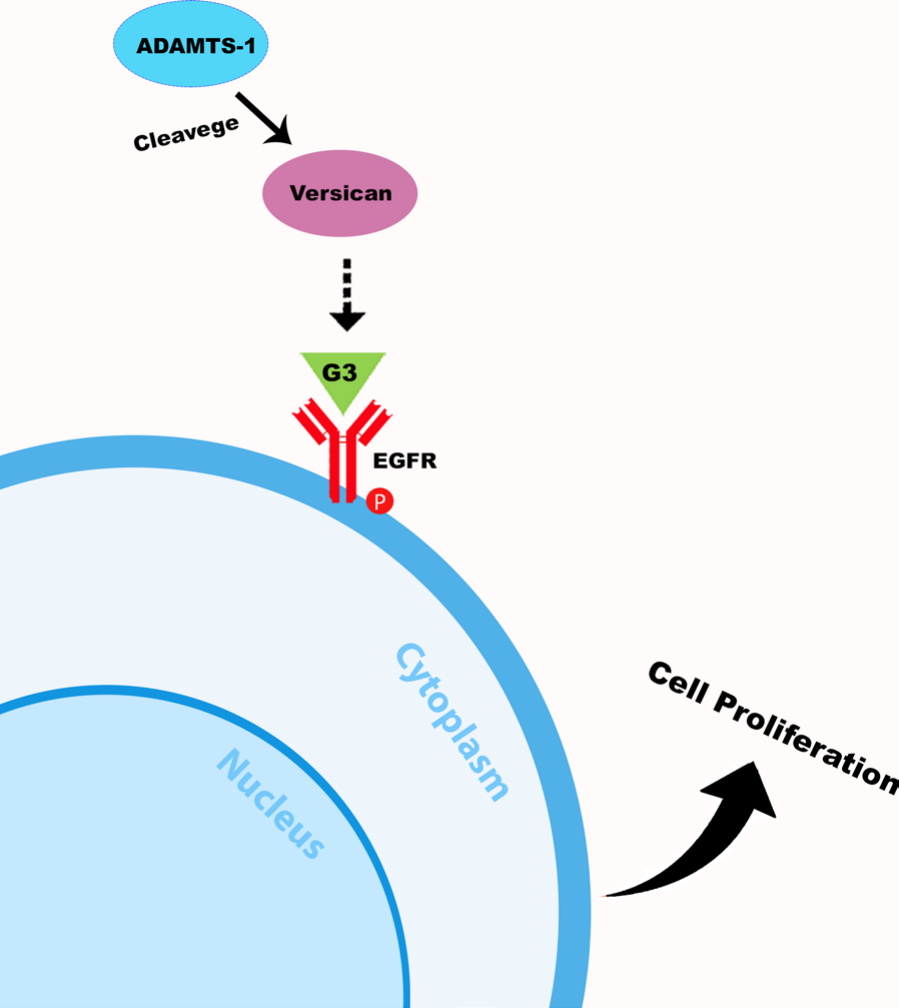
Interaction mechanisms of ADAMTS-1, versican and pEGFR proteins. Author image created in Adobe photoshop version 2019 number 20.0.10

The substantial expressions of ADAMTS-1, versican and pEGFR observed in this study may indicate an important relationship between these proteins and the etiology, pathogenesis and maintenance of the investigated lesions. ADAMTS-1 may have an influence on the cleavage of versican, which releases G3 domains that stimulate the release of EGF-like motifs, which in turn bind to EGFR and contribute to PG and RC evolution (Fig. 4).

Novel available drugs classified as tyrosine kinase inhibitors (such as erlotinib and gefitinib) and monoclonal antibodies (cetuximab) can inhibit or block EGFR expression [25, 26]; thus, we hypothesize that the use of intracanal these kinds of drugs may be a useful tool in the treatment of PG and in particular RC that remain persistent or recurrent after conventional endodontic treatment.

## Conclusions

ADAMTS-1, versican and pEGFR were expressed in the studied lesions. It is known that the pathogenesis of these chronic periapical lesions is characterized by the complex interaction between molecules involved in the immunoinflammatory response process. Despite the methodological limitation of this study, we would like to ask the following question to be answered in a future study. Is there an interaction of the investigated proteins? may it have an influence on the pathogenesis of PG and RC, through the ADAMTS-1 remodeling of the versican ECM that can produce bioactive fragments that activate EGFR and contribute to the formation, growth and maintenance of injuries? Further studies are needed to better support the interaction between the proteins studied and to clarify the pathogenesis of these lesionsd.

**Table 1:** The p-value when comparing the expression of proteins in epithelium among lesions, Kruskal–Wallis test

Lesion	n	ADAM-TS1 (%)			VERSICAN (%)			pEGFR (%)		
		Mean	SD	p-value	Mean	SD	p-value	Mean	SD	p-value
RC	25	87.5	± 6.60		82.1	± 6.58	**	92.2	± 4.86	
IFH	10	89.9	± 4.64		92.5	± 5.14		92.4	± 6.48	
RC	25	87.5	± 6.60	*	82.1	± 6.58	**	92.2	± 4.86	*
DF	10	90.3	± 4.89		89.4	± 5.56		89.2	± 5.44	
IFH	10	89.9	± 4.64		92.5	± 5.14		92.4	± 6.48	*
DF	10	90.3	± 4.89		89.4	± 5.56		89.2	± 5.44	

*RC* radicular cyst, *IFH* inflammatory fibrous hyperplasia, *DF* dental follicle

*p < 0.05; **p < 0.001

**Table 2:** The p-value when comparing the expression of proteins in connective tissue among lesions, Kruskal–Wallis test

Lesion	n	ADAM-TS1 (%)			VERSICAN (%)			pEGFR (%)		
		Mean	SD	p-value	Mean	SD	p-value	Mean	SD	p-value
RC	25	74.6	± 11.59	***	77.6	± 9.66		74.6	± 11.6	***
PG	25	84.5	± 9.69		77.8	± 8.33		84.5	± 9.7	
RC	25	74.6	± 11.59		77.6	± 9.66		74.6	± 11.6	
IFH	10	70.2	± 13.27		78.5	± 9.98		70.2	± 13.3	
RC	10	74.6	± 11.59	***	77.6	± 9.66	***	74.6	± 11.6	***
DF	10	61.7	± 14.48		60,0	± 12.7		61.7	± 14.5	
PG	10	84.5	± 9.69	***	77.8	± 8.33		84.5	± 9.7	***
IFH	10	70.2	± 13.27		78.5	± 9.98		70.2	± 13.3	
PG	10	84.5	± 9.69	***	77.8	± 8.33	***	84.5	± 9.7	***
DF	10	61.7	± 14.48		60.0	± 12.7		61.7	± 14.5	
IFH	10	70.2	± 13.27		78.5	± 9.98	***	70.2	± 13.3	
DF	10	61.7	± 14.48		60.0	± 12.7		61.7	± 14.5	

*RC* radicular cyst,* IFH* inflammatory fibrous hyperplasia,* DF* dental follicle

*p < 0.05; **p < 0.001

## Data Availability

The datasets used and/or analysed during the current study are available from the corresponding author on reasonable request.

## Abbreviations

PG: Periapical granuloma
RC: Radicular cysts
MMPs: Matrix metalloproteinases
ECM: Extracellular matrix
ADAMTS: A Disintegrin and Metalloproteinase with Thrombo Spondin Motifs
EGF: Epidermal growth factor
EGFR: Epidermal growth factor receptor
pEGFR: Phosphorylated epidermal growth factor receptor
IFH: Inflammatory fibrous hyperplasia
DF: Dental Follicle
DAB: Diaminobenzidine
